# Editorial: Women in infectious agents and disease: 2025

**DOI:** 10.3389/fmicb.2026.1884772

**Published:** 2026-06-04

**Authors:** Nayeli Alva-Murillo, Svetlana Khaiboullina, Samira Tarashi

**Affiliations:** 1 Department of Biology, Division of Natural and Exact Sciences, University of Guanajuato, Guanajuato, Mexico; 2 Department of Microbiology and Immunology, University of Nevada, Reno, NV, United States; 3 Department of Mycobacteriology and Pulmonary Research, Pasteur Institute of Iran, Tehran, Iran; 4 Microbiology Research Center (MRC), Pasteur Institute of Iran, Tehran, Iran

**Keywords:** gender equality, human papilloma virus, infectious agents, infectious disease, microbial pathogenesis, microbiome, scientific publishing, women in science

Despite substantial contributions by women to scientific discovery, their achievements remain disproportionately underrecognized at senior academic levels. A 2026 analysis by the International Science Council and partners highlighted that women are markedly underrepresented in leadership roles; with 20% national academy presidents being women (International Science Council et al., 2026). This inequality extends to professional progression, notably affecting women's ability to publish as corresponding authors, with only 17% of submitted manuscripts in “Nature” listing women as corresponding authors (Nature, 2024). Research Topics such as “*Women in Infectious Agents and Disease: 2025*” are therefore important not only for highlighting scientific excellence, but also for creating space where women-led research can gain deserved visibility across the infectious disease field.

The present Research Topic brings together 11 articles, including six original research articles, two reviews, two systematic reviews, and one community case study. Together, these contributions span women's reproductive tract infections, cervical cancer prevention, pregnancy-related disease, surgical infection risk, and fundamental advances in pathogen biology. The collection illustrates both the breadth of women-led infectious disease research and the importance of integrating clinical, microbiological, molecular, and public health perspectives.

A major theme across the collection is the complexity of the female reproductive tract as a dynamic microbial, hormonal, and immunological ecosystem. Among the review articles, De Seta et al. provide new insights into personalized therapies for vulvovaginal candidiasis and vaginal co-infections, emphasizing that vulvovaginal candidiasis and bacterial vaginosis arise not simply from pathogen overgrowth, but from interactions among host immunity, vaginal pH, lactic acid, mucosal responses, and microbiome composition. Their work supports a shift toward individualized strategies aimed at restoring vaginal homeostasis and reducing recurrence. This perspective is extended by Luppi et al., whose systematic review examines how hormonal treatments for endometriosis may influence the reproductive microbiome. Although the available evidence remains limited and heterogeneous, the review suggests that commonly used therapies may promote dysbiotic

shifts in vaginal and endometrial microbial communities, with possible implications for inflammation, fertility, and long-term management. Together, these studies underscore the need to view women's reproductive infectious and inflammatory disorders through an integrated lens that includes microbes, hormones, and host responses.

A second prominent theme is the modernization of cervical cancer prevention through more accessible and precise strategies. In the systematic review and meta-analysis, Ji et al. evaluate self-collected menstrual blood for high-risk human papillomavirus testing and report high pooled sensitivity, though modest specificity, for detecting cervical intraepithelial neoplasia (CIN) and cervical cancer. Their findings support menstrual blood-based testing as a promising adjunct for women who are unable or unwilling to undergo conventional sampling. At the population level, Nemeth Blažić et al. describe the development of a digital interactive cervical cancer risk calculator in Croatia, showing how public-health-hosted digital tools can enhance awareness, health literacy, and engagement with screening and vaccination programs.

This prevention framework is further strengthened by studies that refine biological risk stratification. In an original research article, Li et al. analyze high-risk HPV and bacterial sexually transmitted infections in a rural screening population from Hainan, China, and show that cervical risk is shaped by more than HPV alone. *Ureaplasma urealyticum* emerged as the dominant bacterial pathogen and a central co-infection hub, particularly with HPV52 and HPV58, while *Chlamydia trachomatis* and *U. urealyticum* were differentially associated with early cervical abnormalities. These findings support the value of integrating bacterial STI screening into HPV surveillance in underserved settings. Complementing this work, Yang et al. compare two DNA methylation assays, GynTect^®^ and CISCER^®^, in hrHPV E6/E7 mRNA-positive women and show that both perform comparably in identifying high-grade lesions. Their study highlights methylation-based triage as a useful strategy for improving specificity among women with evidence of active viral oncogene expression. Collectively, these articles point toward a more inclusive and precise model of cervical cancer prevention that combines self-sampling, digital engagement, microbial co-risk profiling, and molecular triage.

Pregnancy-related disease is represented by the study of Ding et al., who examine the association among placental manganese levels, maternal gut microbiota, and preeclampsia. They report significantly lower placental manganese levels in women with preeclampsia and identify bacterial genera associated in opposite directions with manganese and disease status. In particular, *Campylobacter* and *Porphyromonas* were positively associated with preeclampsia and negatively associated with manganese, whereas *Coprobacillus* showed the opposite pattern. Their findings suggest that trace elements and gut microbial ecology may interact in shaping the inflammatory and metabolic environment of preeclampsia, opening new directions for research into pregnancy complications.

The burden of infectious disease in women also extends into surgical and oncologic care. In an original research article, Jarzynka et al. characterize nasal carriage strains of methicillin-sensitive *Staphylococcus aureus* in women undergoing breast reconstruction. Although methicillin-sensitive, these isolates frequently harbored adhesion and biofilm-associated genes and demonstrated strong biofilm-forming ability, highlighting that postoperative infection risk is not defined by methicillin resistance alone. Their findings reinforce the value of preoperative screening and targeted infection prevention in women receiving implant-based reconstruction.

Beyond clinically focused work, this Research Topic also includes fundamental studies of pathogen biology with clear translational relevance. In a review article, Scovino et al. synthesize the molecular mechanisms involved in *Plasmodium* gametocytogenesis, from AP2-G-centered sexual commitment to epigenetic, post-transcriptional, and epitranscriptomic regulation. By focusing on the parasite stage required for mosquito transmission, their work identifies vulnerabilities relevant to transmission-blocking therapies and vaccines for malaria control. At the bacterial level, Berger et al. show that altered *RpoS* expression in *Escherichia coli* O104:H4 profoundly reshapes global gene expression and carbon source utilization, illustrating how nutrient adaptation, stress responses, and virulence are tightly interconnected in pathogenic bacteria. Finally, Alba et al. report the first documented case of *Mycobacterium africanum* lineage 6 infection in a cat, supported by genomic characterization and phylogenetic analysis. This unusual finding expands the known host range of the pathogen and highlights the importance of genomics and One Health perspectives in tuberculosis surveillance and understanding transmission.

Overall, this Research Topic demonstrates that supporting women-led research is not only a matter of equity, but also a driver of scientific progress. The contributions collected here advance understanding across public health, diagnostics, microbial ecology, pregnancy complications, pathogen genomics, and molecular pathogenesis. They also show that progress in infectious disease research increasingly depends on integrating perspectives across scales—from the vaginal microenvironment and cervical screening pathway to the placenta, the surgical ward, and the molecular circuitry of globally important pathogens. By providing an inclusive platform for women investigators, this Research Topic helps strengthen visibility, recognition, and career development while enriching the field with diverse, high-quality science.
